# Effects of Halotherapy as an Adjunct to Preseason Training on Respiratory and Aerobic Outcomes in Elite Female Football Players: A Randomized Controlled Trial

**DOI:** 10.3390/life16081233

**Published:** 2026-07-25

**Authors:** Derya Çetin Sarışık, Ahmet Serhat Aydın, Mehmet Söyler, Raif Zileli, Ali Özkurt, Coskun Yılmaz, Hamza Küçük, Mevlüt Karataş

**Affiliations:** 1 Faculty of Sport Sciences, Bayburt University, Bayburt 69000, Türkiye; deryacetin@bayburt.edu.tr; 2 Institute of Health Sciences, Gazi University, Ankara 06560, Türkiye; serhataydingazi@gmail.com; 3 Vocational School of Social Sciences, Cankiri Karatekin University, Cankiri 18200, Türkiye; mehmetsoyler@karatekin.edu.tr; 4 Department of Child Development, Faculty of Health Science, Bilecik Şeyh Edebali University, Bilecik 11000, Türkiye; raif.zileli@bilecik.edu.tr; 5 Clinic of Internal Medicine, Çankırı Private Karatekin Hospital, Cankiri 18100, Türkiye; drozkurt@gmail.com; 6 Faculty of Sports Sciences, Gumushane University, Gumushane 29000, Türkiye; 7 Yasar Dogu Faculty of Sports Sciences, Ondokuz Mayis University, Samsun 55270, Türkiye; 8 Department of Chest Diseases, Ankara Atatürk Sanatorium Training and Research Center, University of Health Sciences, Ankara 06290, Türkiye; mevlutkaratas@karatekin.edu.tr; 9 Rectorate, Cankiri Karatekin University, Cankiri 18200, Türkiye

**Keywords:** halotherapy, elite female football players, respiratory health, aerobic performance, training intervention

## Abstract

Background: Aerobic capacity and respiratory efficiency are critical determinants of performance in elite female football, characterized by repeated high-intensity efforts and rapid recovery demands. Halotherapy, involving the inhalation of dry salt aerosol, has been shown to improve pulmonary health; however, its effects on exercise-related outcomes in athletic populations remain insufficiently investigated. This study aimed to examine the effects of a 12-week post-training halotherapy intervention on respiratory muscle strength, pulmonary function, and aerobic performance in elite female football players. Methods: Twenty-eight elite female football players competing in the Turkish Women’s First League were randomly assigned to a halotherapy group (HG, *n* = 14) or a control group (CG, *n* = 14). Both groups completed an identical preseason training program, while the HG additionally performed 45 min halotherapy sessions three times per week in a controlled halochamber. Pre- and post-intervention assessments included maximal inspiratory and expiratory pressures (MIP, MEP), spirometric parameters (FVC, FEV_1_, PEF, MVV), and maximal oxygen uptake (VO_2_max) estimated using the Yo-Yo Intermittent Recovery Test Level 1 (Yo-Yo IRT1), along with total distance covered. A 2 × 2 mixed-model ANOVA was used to assess time and group × time interaction effects. Results: Significant group × time interactions were observed for MIP, MEP, VO_2_max, FEV_1_, PEF, MVV, and Yo-Yo IRT1 performance (*p* < 0.05), with large effect sizes (η*p*^2^ = 0.269–0.924), favoring the halotherapy group. Forced vital capacity (FVC) increased over time in both groups (*p* = 0.141), with no significant interaction effect. Changes in the control group were comparatively smaller across most outcomes. Conclusion: Post-training halotherapy was associated with improvements in respiratory muscle strength, ventilatory function, aerobic capacity, and intermittent running performance in elite female football players. These findings suggest that halotherapy may be considered as a complementary, non-invasive strategy to support respiratory and aerobic adaptations. Further research is required to confirm these findings and to clarify the underlying physiological mechanisms. Trial registration: NCT07406386; 6 February 2026.

## 1. Introduction

Aerobic capacity and respiratory efficiency are critical determinants of performance in elite female football, particularly given the repeated high-intensity nature of match play and the limited recovery time between efforts [1,2,3,4]. The rapid growth of women’s football over recent decades, both in participation and competitive standards, has further increased the need to better understand the physiological and performance-related demands of female players [5,6]. Consequently, scientific research has increasingly focused on identifying and optimizing key determinants of performance in female football athletes [7].

Elite female football players typically cover distances ranging from 5480 to 11,160 m per match, reflecting substantial endurance demands [4]. Match play is also associated with high cardiovascular strain, with average heart rates reaching approximately 86% of maximum and VO_2_max values reported between 77 and 80% of maximal capacity [1,2]. These demands highlight the importance of aerobic metabolism not only for sustaining performance but also for facilitating recovery during repeated high-intensity efforts [3]. Accordingly, improving aerobic capacity remains a central objective in football training programs [7,8]. While traditional conditioning protocols primarily target cardiovascular and muscular adaptations, increasing attention has been directed toward complementary, non-invasive strategies that may further support respiratory function and aerobic performance. Among these approaches, halotherapy has emerged as a potential intervention.

Recent systematic reviews have demonstrated that respiratory interventions, particularly inspiratory muscle training (IMT), can improve respiratory muscle strength, ventilatory efficiency, and exercise performance in athletic populations. However, the reported effects on pulmonary function, aerobic capacity, and sport-specific performance remain heterogeneous and appear to depend on participant characteristics, training status, intervention duration, and methodological quality. Similarly, several recovery modalities, including cryotherapy, cold-water immersion, compression garments, and photobiomodulation, have been widely investigated to accelerate post-exercise recovery.

Although halotherapy has gained increasing popularity among athletes, comprehensive research on its efficacy remains limited. Current evidence suggests that halotherapy may have potential as an ergogenic aid; however, findings across different athletic populations, particularly elite female football players, remain scarce. Consequently, further randomized controlled trials are needed to clarify its specific physiological and performance-related effects [9,10].

Halotherapy, or salt therapy, involves inhaling a dry salt aerosol in a controlled environment designed to mimic the conditions of natural salt caves [11,12]. It has been widely applied in clinical settings to improve pulmonary function and alleviate respiratory symptoms [13,14]. The proposed mechanisms underlying these effects include anti-inflammatory, antibacterial, and mucociliary clearance processes that may enhance airway function [15,16]. Additionally, halotherapy has been investigated as a potential adjunctive intervention for respiratory conditions such as asthma and chronic obstructive pulmonary disease; however, the available evidence remains limited, and further high-quality randomized controlled trials are required [17,18,19].

Existing literature on the effects of halotherapy on sports performance remains limited, with studies such as Catalina et al. [20] indicating that a 21-day intervention significantly improves pulmonary functions, including vital capacity and maximum ventilation, in young middle-distance runners. These findings highlight a potential ergogenic aid, yet comprehensive research on diverse athletic populations is still needed.

For athletes, improvements in respiratory function may enhance oxygen delivery and utilization during exercise, potentially supporting both performance and recovery. Previous studies have also suggested that halotherapy may aid recovery processes by reducing muscle soreness and fatigue [21]. However, despite these promising findings, research examining the effects of halotherapy in athletic populations remains limited. In particular, evidence regarding its impact on aerobic capacity and sport-specific performance in team sports such as football is scarce. The studies are particularly relevant to geriatric individuals, children with bronchial asthma, or ankylosing spondylitis, rather than team sports like football [9,16,22,23,24].

Despite the growing body of research investigating respiratory interventions and halotherapy, female athletes remain markedly underrepresented in this field. Most previous studies have included male participants or mixed-sex samples, limiting the ability to extrapolate findings to elite female football players. Furthermore, female athletes differ from males in several physiological characteristics relevant to respiratory function and exercise performance, including lung size, ventilatory responses, hormonal milieu, and aerobic adaptations. Although it remains unclear whether these biological differences influence the effectiveness of halotherapy, they justify the need for sex-specific investigations. Therefore, evaluating halotherapy specifically in elite female football players addresses an important gap in the current literature and contributes evidence from a population that has been relatively neglected in previous research.

Therefore, the present study aimed to investigate the effects of a 12-week post-training halotherapy intervention, simulating a salt-cave environment, on respiratory parameters and aerobic endurance in elite female football players competing in the Turkish First League. Given that aerobic capacity is a key determinant of football performance [25,26], evaluating the potential contribution of halotherapy to these outcomes may provide practical insights for athletes, coaches, and sports science practitioners. It was hypothesized that adding halotherapy to a standard preseason training program would result in greater improvements in respiratory function and aerobic performance than training alone.

## 2. Materials and Methods

**Participants**

Twenty-eight female football players from a single professional team competing in the Turkish Women’s Football First League voluntarily participated in the study. Participants were classified as elite female football players because they competed in the highest national league in Türkiye, trained regularly under professional coaching supervision, and participated in structured preseason and competitive programs. The classification was therefore based on competitive level rather than solely on physiological characteristics. The sample was divided into two groups: Control Group (CG, *n* = 14; age: 21.93 ± 2.13 years; height: 169.86 ± 5.30 cm; weight: 59.96 ± 3.34 kg) and Halotherapy Group (HG, *n* = 14; age: 23.14 ± 2.63 years; height: 165.96 ± 5.09 cm; weight: 62.63 ± 3.02 kg). Participants were selected through convenience sampling (Table 1).

Inclusion criteria included: age between 18 and 30 years, at least 5 years of competitive football experience, regular training exceeding 10 h per week, no current injuries or medical conditions that could affect participation, non-smokers not on chronic medication, and not pregnant. All participants provided written informed consent in accordance with the Declaration of Helsinki. The study protocol was approved by the Ethics Committee of Bilecik Şeyh Edebali University (Approval No: 2022/2-3). Power analysis (G*Power 3.1.9.7) indicated a minimum sample size of 20 to detect an effect size of 0.35 with 80% power at α = 0.05 for repeated measures ANOVA [27].

Participants were randomly allocated to the halotherapy group or control group using a computer-generated randomizer (https://www.randomizer.org/) random sequence created by an investigator who was not involved in outcome assessment or statistical analysis. Allocation assignments were concealed in sequentially numbered, opaque, sealed envelopes until baseline testing had been completed. Because the intervention involved attendance in a halotherapy chamber, participant blinding was not feasible. However, outcome assessments were performed by investigators who were not involved in group allocation whenever possible, and statistical analyses were conducted using coded datasets.

**Study design**

The study was a randomized parallel-group trial over 14 weeks during the 2022–2023 pre-season. Participants were randomized to either the CG or HG. The intervention combined halotherapy with regular training for the HG, while the CG completed training alone.

**Figure 1:**
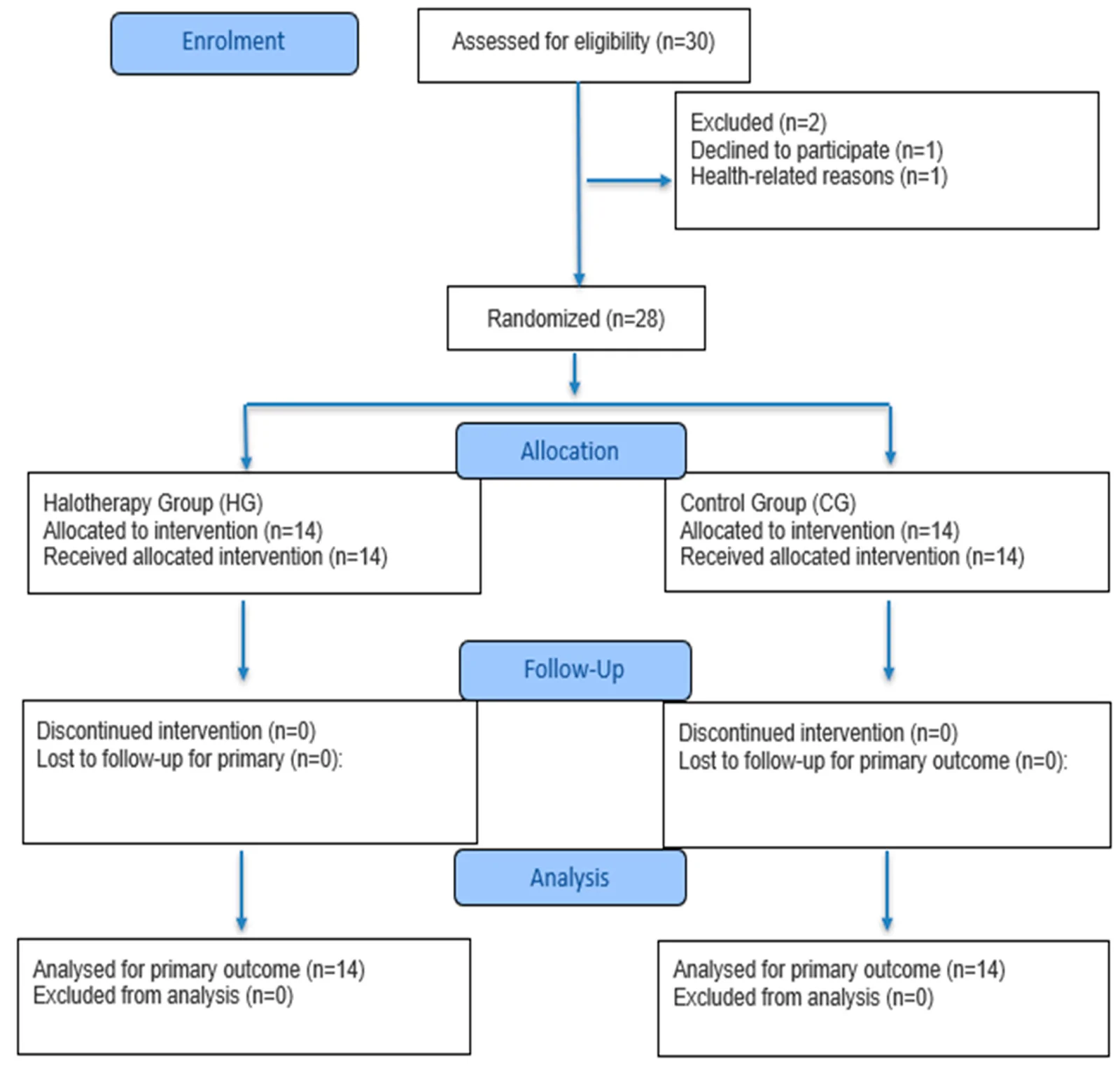
Experimental design.

The current study employed a randomized parallel-group design to compare two groups: the CG and the HG. The treatment group participated in sessions conducted in halochambers, artificial salt-cave rooms, as described by Chervinskaya [28]. Each group comprised fourteen athletes, and participants were randomly assigned to the control or treatment group to ensure unbiased allocation. The study was conducted during the pre-season period of the 2022–2023 season and spanned 14 weeks. This duration encompassed a week dedicated to pre-testing, followed by twelve weeks of training, and finally, a week designated for post-testing (Table 2 and Figure 1).

**Training Regimen**

Both groups participated in the team’s standard training program, which included endurance, technical/tactical drills, and strength-training components (Table 3). Strength training was conducted in a gymnasium under the supervision of conditioning coaches.

**Training Load Monitoring**

Internal training load was monitored throughout the 12-week intervention using the session Rating of Perceived Exertion (sRPE) method based on the CR-10 Borg scale. Approximately 30 min after each training session, players individually reported their perceived exertion, and the values were recorded by the conditioning staff. Training intensity was generally maintained within a target range of 5–7 arbitrary units (AU), corresponding to moderate-to-vigorous exercise intensity. Both groups followed the same preseason football training programme, supervised by the same coaching staff, with identical session frequency, duration, content, and progression. Attendance was monitored throughout the intervention period, and no meaningful differences in training exposure were observed between the halotherapy and control groups. The only between-group difference was the addition of halotherapy sessions in the intervention group (Table 4).

**Intervention Protocol**

The HG underwent 45 min sessions in a halochamber three times per week before their regular 90 min football training. Sessions were conducted in the team’s training camp locker room, minimizing travel and maximizing adherence. Over 12 weeks, participants completed 36 halotherapy sessions. The CG trained as usual without halotherapy (Table 5).

**Testing Procedures**

Testing Schedule

All outcome assessments were performed under standardized laboratory conditions at approximately the same time of day. During the study, the pre- and post-tests were administered on Wednesdays in the first and last weeks. The choice of these weekdays ensured that the assessment occurred under similar conditions for both the pre-intervention and post-intervention evaluations. During the first preseason visit, which included the pre-intervention assessment, several measurements were taken in the morning (between 09:00 and 10:00). These included body mass, height, body mass index, and a respiratory function test. Later in the afternoon (around 16:00), the Yo-Yo IRT1 (VO_2_max) intermittent fitness performance value was determined during the same first pre-season visit. To ensure consistency and eliminate potential confounding factors, athletes were instructed not to alter their diets on the pre- and post-test days, to avoid strenuous exercise 24 h before the test, to refrain from consuming caffeinated drinks at least 12 h before each assessment, to maintain their normal fluid intake, and to get a normal night’s sleep before the test. Menstrual cycle phase was not controlled or recorded during testing; therefore, a potential influence of hormonal fluctuations on the measured physiological outcomes cannot be excluded.

**Pre-Test Preparation**

All participants completed a standardized 20 min warm-up before testing. Instructions before testing included: Voiding the bladder 30 min prior, avoiding heavy meals 3 h prior, and abstaining from strenuous exercise 12 h prior.

**Warm-Up Protocol**

All participants followed a standardized warm-up protocol before performance testing and regular training sessions. The objective of this routine was to facilitate physiological readiness by activating the musculoskeletal and cardiorespiratory systems, enhancing neuromuscular coordination, and minimizing the risk of injury [29,30]. The entire protocol was structured in three progressive stages and lasted approximately 20 min.

**Aerobic Initiation (5–6 min)**

The warm-up began with low- to moderate-intensity aerobic movements intended to elevate core body temperature and gradually increase heart rate. Exercises in this phase included: light jogging, lateral shuffles, high knees, butt kicks, and crossover steps (karaoke drill). This phase aimed to increase cardiovascular output and initiate central activation while maintaining low mechanical demand [31].

**Dynamic Mobility (6–8 min)**

The second stage focused on dynamic stretching and mobility drills targeting major muscle groups and joint structures. Athletes performed the following movements over 10–15 m distances: hip openers and leg swings (sagittal and frontal planes), walking lunges with torso rotation, lateral lunges, ankle mobilization drills, arm circles, and dynamic shoulder swings.

Unlike static stretching, dynamic mobility was preferred due to its positive effects on movement efficiency and acute performance outcomes [32].

**Neuromuscular Activation and Sport-Specific Movements (5–6 min)**

The final phase incorporated high-intensity drills to elicit neuromuscular responsiveness and simulate game-like demands. This included: acceleration sprints over short distances (3 × 10 m), vertical and horizontal jumps, change-of-direction drills, ball handling with rapid passes and sprints. These movements were designed to increase motor unit recruitment and improve reaction speed and agility in sport-specific contexts, particularly in intermittent, high-intensity sports such as football [33].

**Environmental Conditions and Monitoring**

Warm-up sessions were conducted on the same synthetic turf as the training ground, under controlled environmental conditions (20–24 °C, 40–60% humidity). All athletes wore standard training kits and appropriate footwear (cleats). The intensity of the warm-up was subjectively monitored using the Rating of Perceived Exertion (RPE) scale and maintained at a moderate level (RPE 3–5), in accordance with Foster et al. [34].

**Anthropometric and Body Composition measurements**

An electronic measuring device was used to measure height in the anatomical position, with participants barefoot and ensuring that their body weight was evenly distributed on both feet. The distance from the top of their head to the floor was measured in meters (m) with an accuracy of 0.1 mm. To accomplish this, a height scale manufactured by Seca (Germany) was utilized, following the methodology established by Miller et al. [35]. The process of assessing the players’ body composition involved several steps. First, height was accurately measured, and individuals with recorded height were selected for body composition analysis. The measurements included body weight (in kilograms) and body mass index (BMI). Bioelectrical impedance analysis (BIA) was performed using the InBody 270 Body Composition Analyzer (model Plus 270). BIA is a method that relies on the distinction in electrical conductivity [36]. To ensure consistency, body composition measurements were conducted between 09:00 and 10:00 the day before the performance tests. The participants were instructed to fast and to abstain from any food or liquids after dinner, and they were assisted in using the restroom before the measurements. During the analysis procedure, the football players were asked to remove any metallic or decorative accessories they were wearing. They were lightly dressed and positioned upright, barefoot, while grasping the hand electrodes connected to the analysis device’s aluminum platforms. The data obtained during the body composition analysis was accurately recorded through the computer, which was connected to the body composition analyzer [37].

**Pulmonary Function Tests**

Spirometry was conducted using Spirolab III (Medical International Research, Rome, Italy) in a controlled environment (20–22 °C, 45–55% humidity) by the same researcher. To determine lung volumes and capacities in football players, forced vital capacity (FVC), and forced expiratory volume in 1 s (FEV1) were measured using a spirometer. The Spirolab III Spirometer (Medical International Research, Rome, Italy) was used for these measurements. Measurements were made simultaneously in the same room at similar temperatures by a single researcher. The test procedure consisted of the following steps: players received verbal instructions, and their age, height, and gender were recorded for accurate data analysis. The spirometry tests were conducted in a controlled sports hall with stable temperature and pressure. The players were seated in an upright position on a chair with back support. To ensure accurate measurements, the players were instructed to securely place the spirometer mouthpiece in their mouths to prevent air leakage. The nostrils were occluded with a clip to prevent air from escaping through the nose. Forced Vital Capacity Measurement (FVC): Players were instructed to perform a forceful, rapid exhalation, starting with maximal inspiration, and to continue for at least 6 s. The best FVC value was determined, ensuring that the difference between the best FVC values was less than 200 mL. Forced Expiratory Volume in 1 Second Measurement (FEV1): In this stage, FEV1 parameters were measured. The players took a normal breath for 5 s, followed by a rapid, forceful inspiration, and then a rapid expiration for 6 s or until a flat plateau was observed on the volume-time curve [38]. Pulmonary function testing was performed in accordance with the latest American Thoracic Society/European Respiratory Society (ATS/ERS) Technical Standard for Spirometry. All spirometric measurements were conducted by a trained examiner using standardized procedures.

**Maximal inspiratory (MIP) and expiratory (MEP) pressure measurements**

The MIP and MEP were measured with a portable hand-held oral respiratory pressure meter (MicroRPM, CareFusion Micro Medical, Kent, UK) according to the guidelines of the American Thoracic Society and European Respiratory Society [39]. After the appropriate filters and holders were secured, the nasal airway was closed with a clip. The mouthpiece assembly included a 1 mm hole to prevent glottic closure and minimize the contribution of the cheek muscles during inspiratory efforts. Inspiratory and expiratory maneuvers were performed while standing, with MIP and MEP measurements starting with residual volume and total lung capacity, respectively, and continued for at least 1 s. The measurements were repeated until there was a 5% difference between the two best findings, and the results were recorded in cm H_2_O [38].

**Yo-Yo Intermittent Recovery Test Level 1**

This test included 2 × 20 runs, performed at a gradually accelerated speed and controlled by beeping sounds from an iPad (Apple Inc., New York, NY, USA) connected to a Bluetooth speaker (Xiaomi Inc., MDZ, Tokyo, Japan) located immediately next to the 20 m long finishing line. Each participant had a 10 s rest before running to the next cone, 5 m beyond the point at which athletes needed to turn and head toward the starting line. The test concludes when the participant either stops due to exhaustion or fails to step over the line before the beep sounds on two consecutive occasions. Four runs were performed at 10–13 km·h^−1^, and another seven runs were at 13.5–14 km·h^−1^. Then, after completing every eight runs and until they were fatigued, participants had speed increments of 0.5 km·h^−1^ [40]. During this test, the participant’s performance is evaluated based on the total distance covered, regardless of whether they completed the entire stage [24]. The reliability of the Yo-Yo IRT1 test has been established in a previous study, showing an Intra-Class Correlation Coefficient (ICC) of 0.98 and a coefficient of variation (CV) of 3.5%. These values indicate a high level of reliability and consistency in the test results [38].

Estimated maximal oxygen uptake (VO_2_max) was calculated from the Yo-Yo Intermittent Recovery Test Level 1 (Yo-Yo IRT1) distance using the equation proposed by Bangsbo et al. [41]: VO_2_max (mL·kg^−1^·min^−1^) = IR1 distance (m) × 0.0084 + 36.4.

**Halotherapy Facility and Procedure**

The halochamber, located in the team’s training camp locker room, was equipped with the Dry Salt Air HaloCompact-01 halogenerator (Halogen UAB, Vilnius, Lithuania), which dispersed micronized dry salt particles (<5 μm) at 20 mg/m^3^ concentration. The chamber temperature and humidity were maintained at 20–24 °C and 40–60%, respectively (Table 6 and Figure 1). Halotherapy sessions were conducted immediately before each scheduled football training session throughout the intervention period. The pre-training administration was standardized for all participants to ensure uniform intervention delivery and to minimize potential confounding effects related to post-exercise fatigue or recovery. After completing the halotherapy session, participants immediately participated in the planned football training programme under identical coaching supervision.

**Statistical Analysis**

The data analysis was performed using SPSS 26.0 (SPSS, Inc., Chicago, IL, USA). The normality of the numerical variables was assessed using the Shapiro–Wilk test, and all variables were found to have a normal distribution (*p* > 0.05). To compare measurements, a 2 × 2 mixed ANOVA (Mixed Variance Analysis) was conducted to assess whether there were significant differences between study groups and test times for pre-test and post-test measurements in the HG and CG. In cases where a significant difference was found, pairwise comparisons were analyzed using the Bonferroni test. The effect size was evaluated using partial eta squared (η*p*^2^), which indicates the proportion of variance in the dependent variable explained by a specific independent variable. Effect sizes were interpreted using the commonly adopted thresholds for partial eta squared (η*p*^2^): 0.01 as small, 0.06 as medium, and 0.14 as large. The results were assessed using a 95% confidence interval, and *p*-values < 0.05 were considered statistically significant.

## 3. Results

Group × time interaction effects for respiratory muscle strength, pulmonary function, and aerobic performance are presented in Table 7.

**Figure 2:**
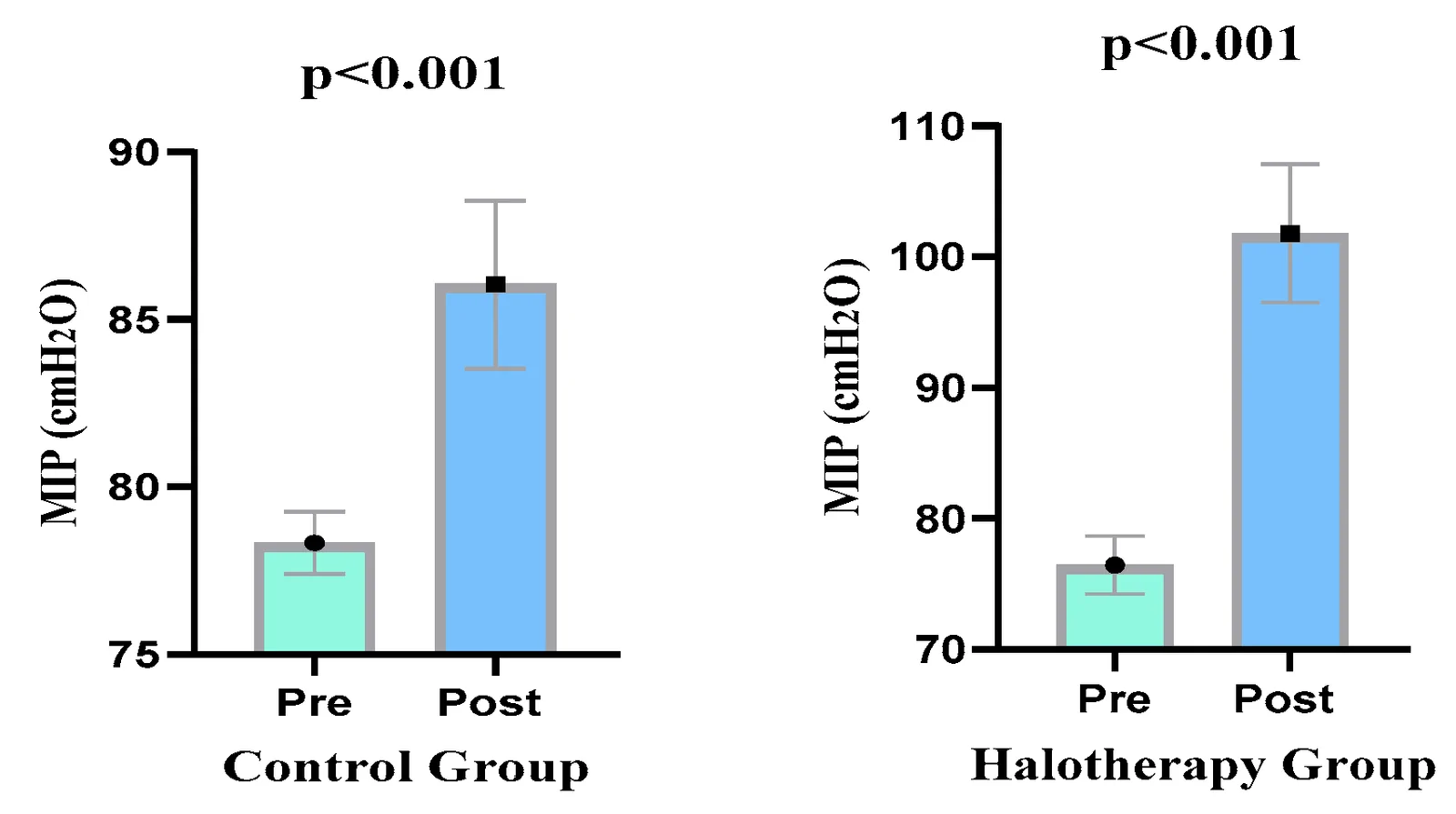
Pre- and post-test changes in MIP performance.

**Figure 3:**
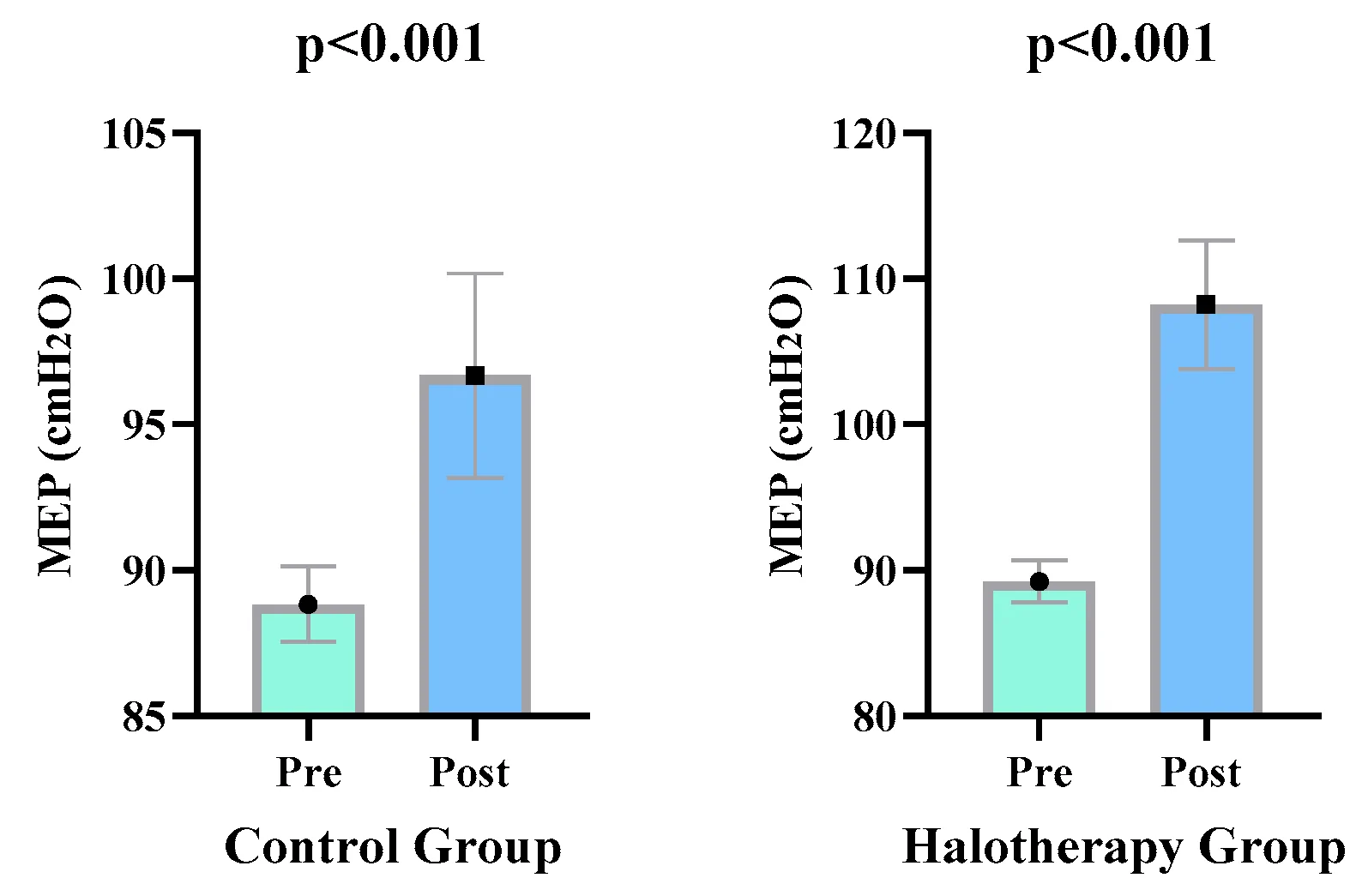
Pre- and post-test changes in MEP performance.

**Figure 4:**
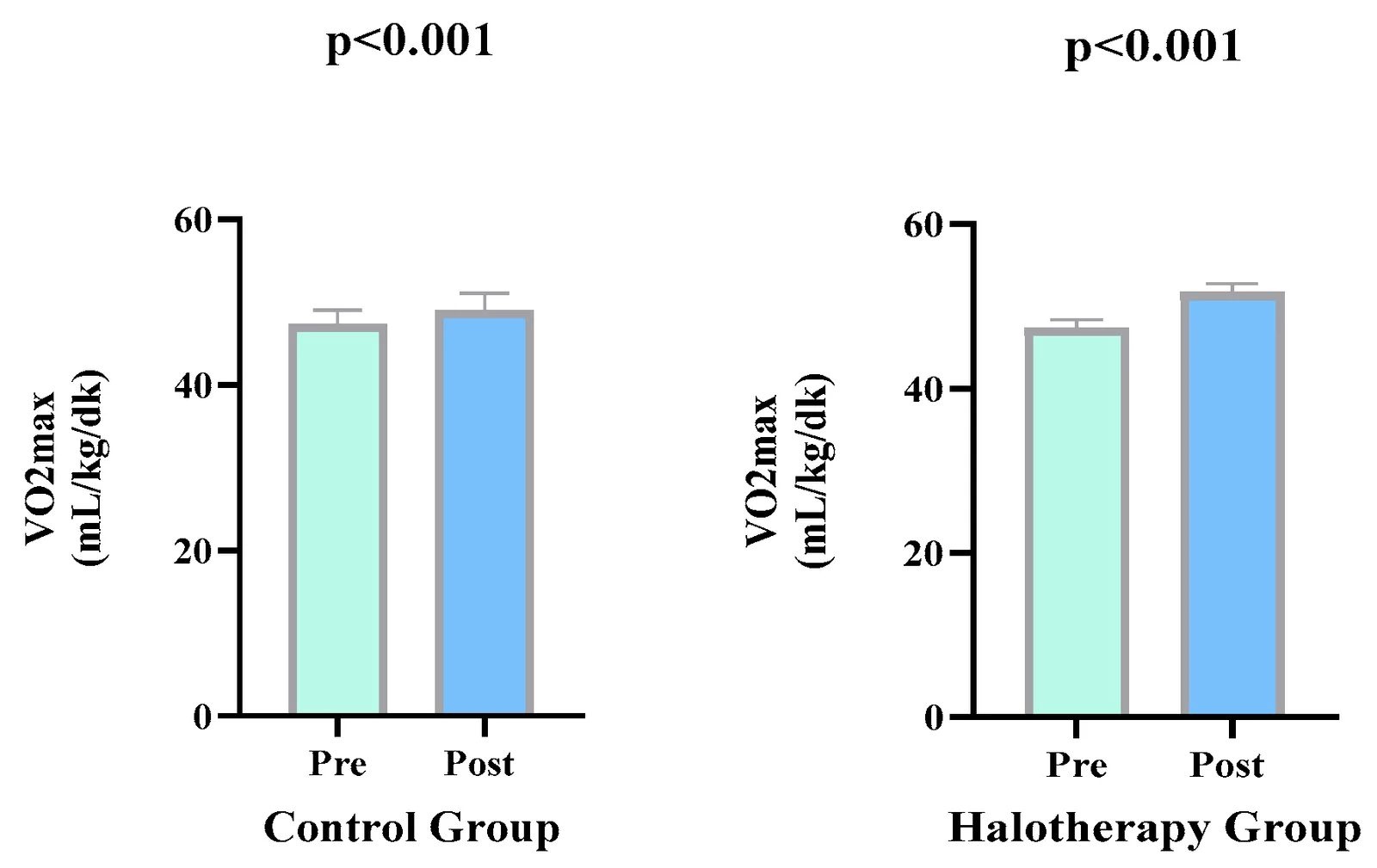
Pre- and post-test changes in VO_2_max performance.

**Figure 5:**
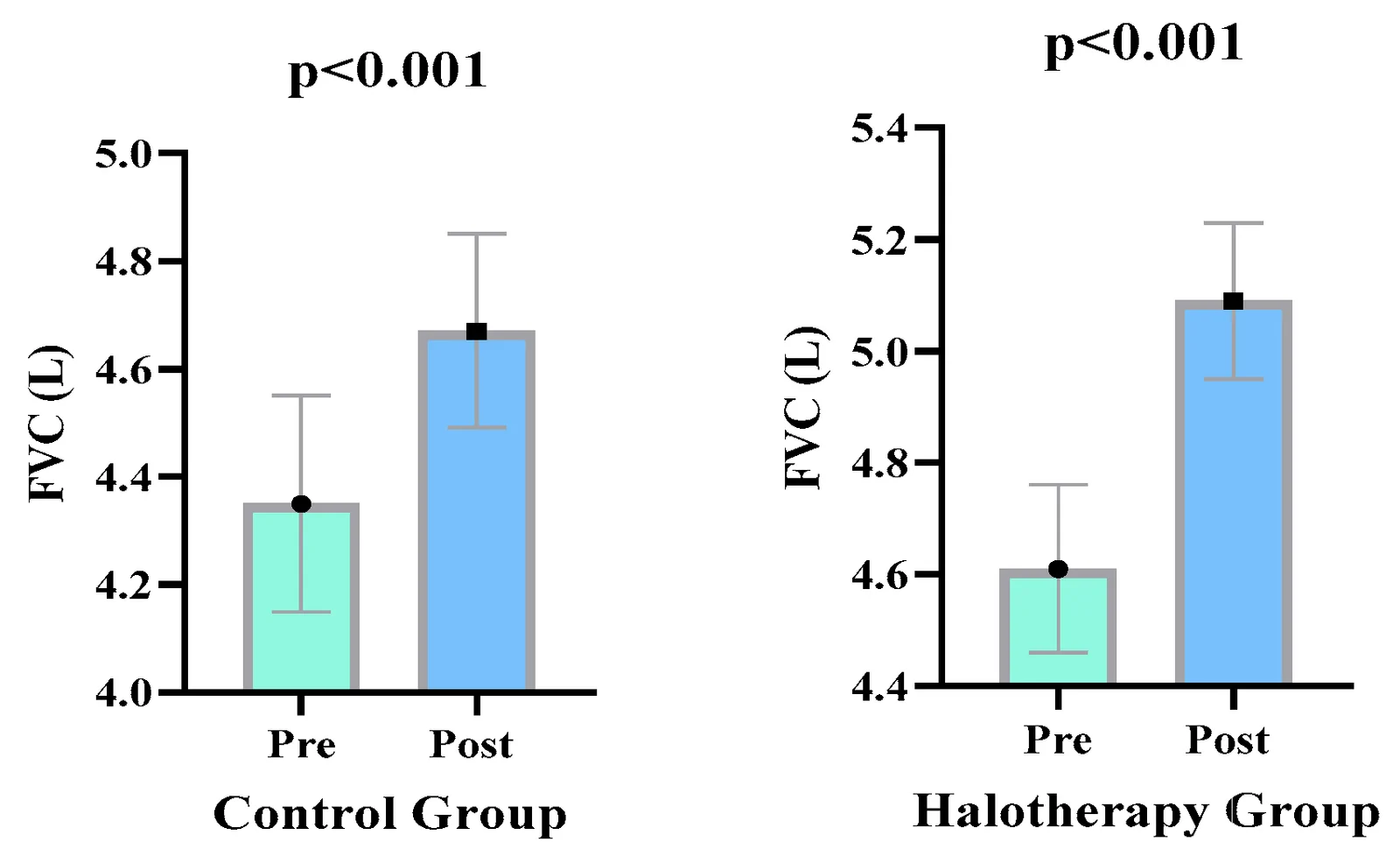
Pre- and post-test changes in FVC performance.

**Figure 6:**
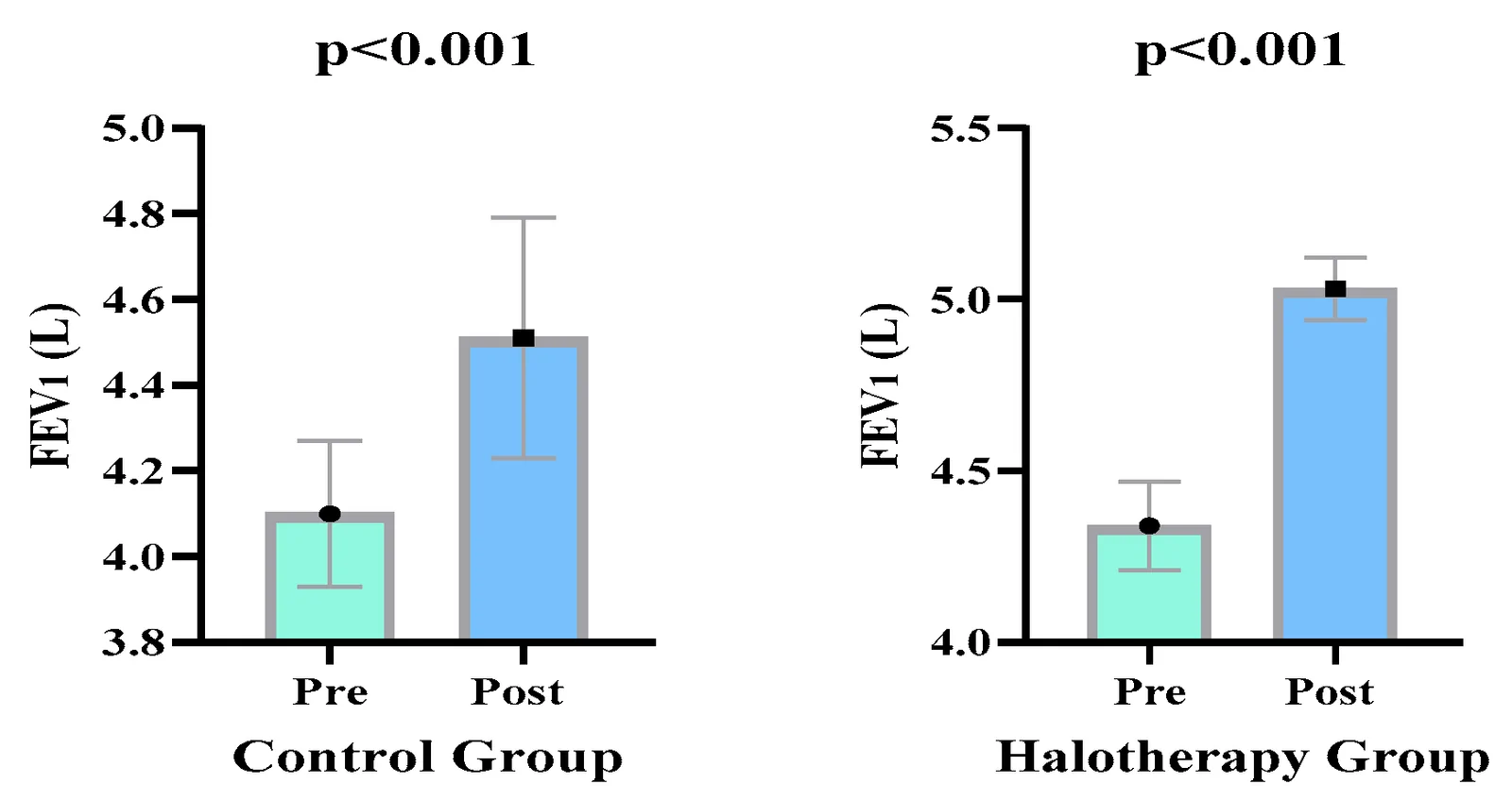
Pre- and post-test changes in FEV_1_ performance.

**Figure 7:**
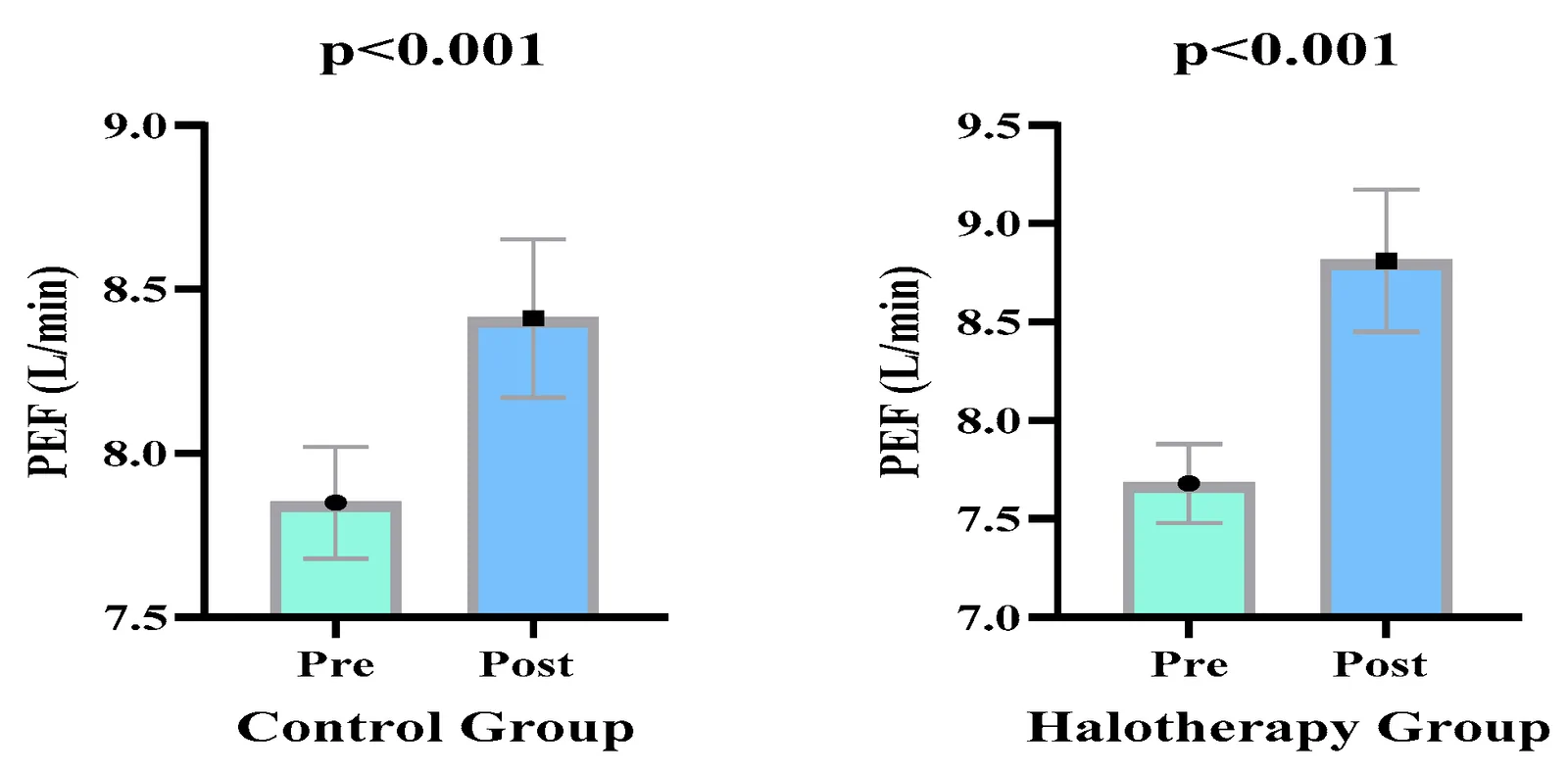
Pre- and post-test changes in PEF performance.

**Figure 8:**
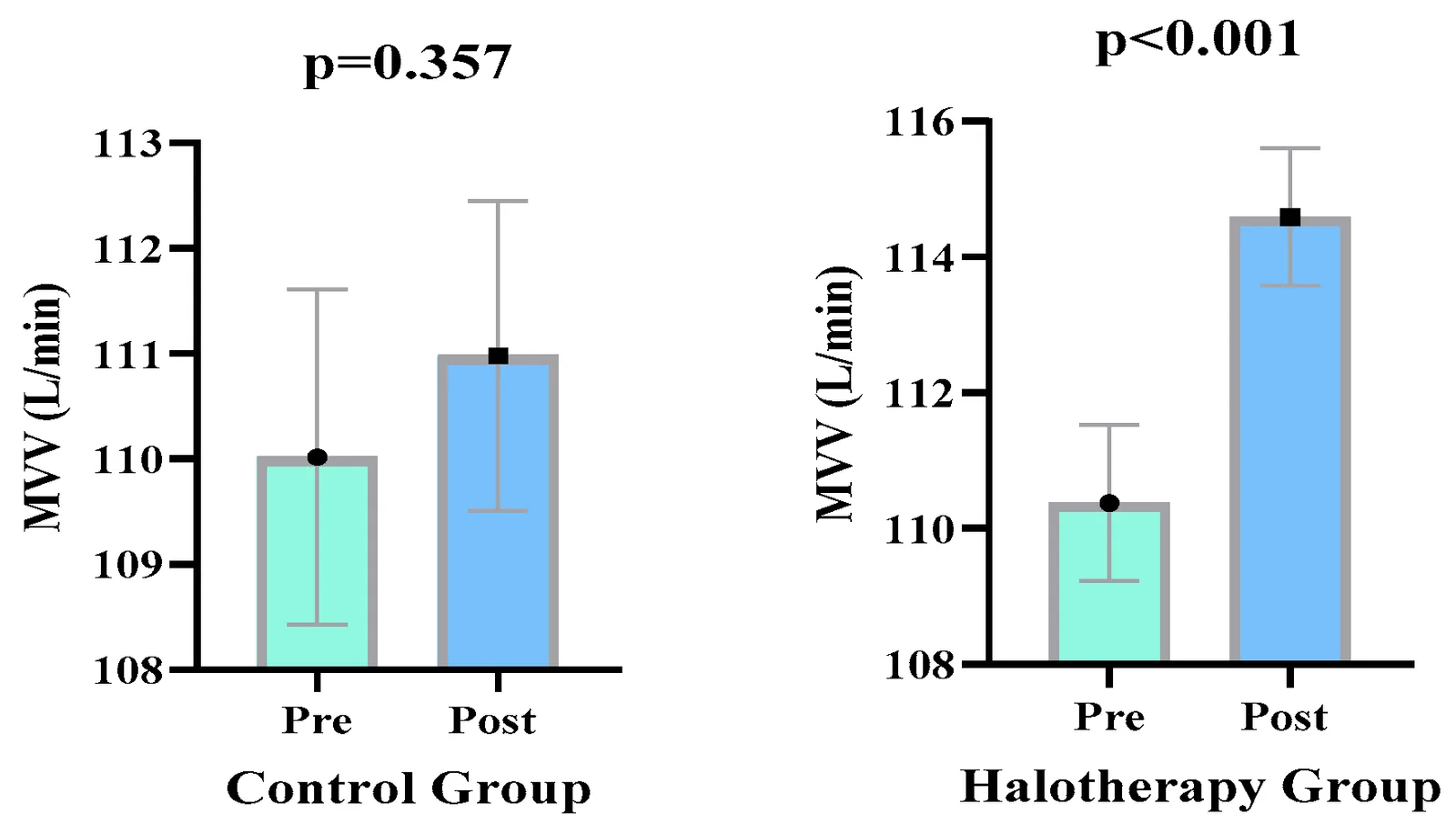
Pre- and post-test changes in MVV performance.

**Figure 9:**
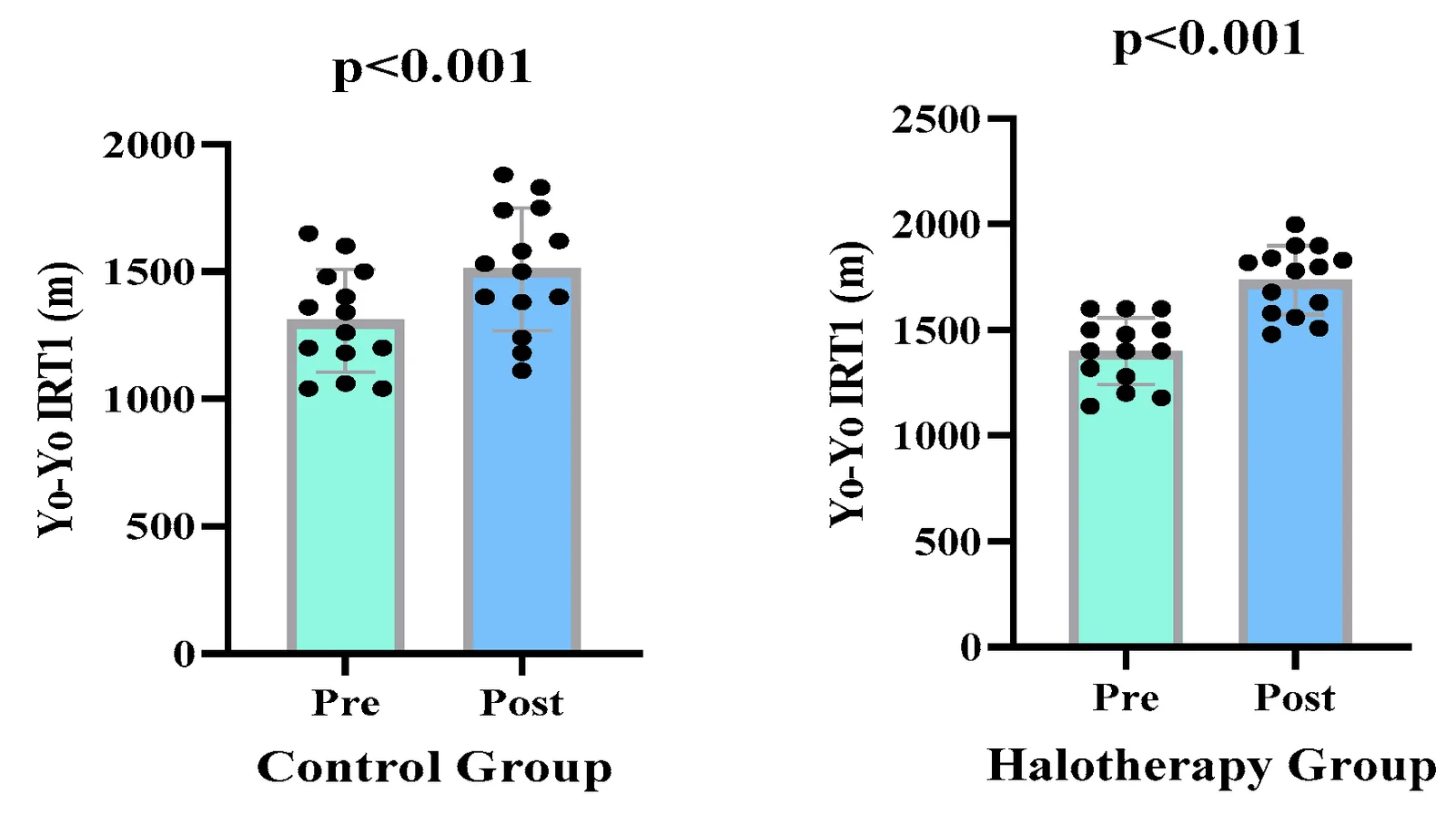
Pre- and post-test changes in Yo-Yo IRT1(m) performance.

Table 7 and Figure 2, Figure 3, Figure 4, Figure 5, Figure 6, Figure 7, Figure 8 and Figure 9 show the changes between pre-test and post-test measurements in the experimental (HG) and control (CG) groups, as well as the time effect and group × time interactions. The findings reveal that the intervention had a significant and large effect size on respiratory muscle strength, pulmonary function, and aerobic performance.

In terms of maximum inspiratory pressure (MIP), an increase was observed over time in both groups; however, the increase in the HG (33.11%) was significantly higher than that in the CG (9.85%). The time effect was statistically significant (F = 217.522, *p* < 0.001, η*p*^2^ = 0.924), and the group × time interaction was also found to be significant (F = 61.790, *p* = 0.014, η*p*^2^ = 0.774). This result indicates that the intervention is highly effective in increasing inspiratory muscle strength (Table 7 and Figure 2).

Maximal expiratory pressure (MEP) values increased in both groups after the intervention; however, the magnitude of the increase was significantly greater in the experimental group (HG) compared to the control group (CG). In the HG, the MEP value rose from 89.23 ± 1.44 cmH_2_O to 108.21 ± 4.41 cmH_2_O, representing a 21.27% increase, while in the CG, the increase was limited to 8.82%. Repeated measures analysis revealed that the time effect was statistically significant (F = 23.544, *p* < 0.001, η*p*^2^ = 0.523), indicating that the overall increase in MEP values reflected a time-dependent adaptation. However, the group × time interaction was also found to be significant (F = 6.857, *p* < 0.001, η*p*^2^ = 0.341), indicating that the increase observed in the experimental group was intervention-specific and more pronounced compared to the control group (Table 7 and Figure 3).

A significant increase of 9.04% in VO_2_max values was observed in the HG, while this increase was limited in the CG (3.57%). The time effect was strong and significant (F = 136.569, *p* < 0.001, η*p*^2^ = 0.840), and the group × time interaction was also statistically significant (F = 25.535, *p* < 0.001, η*p*^2^ = 0.495). This finding suggests that the intervention produced a clinically meaningful improvement in cardiorespiratory fitness (Table 7 and Figure 4).

Forced vital capacity (FVC) increased in both groups (HG: 10.41%; CG: 7.35%). Although the time effect was significant (F = 59.259, *p* < 0.001, η*p*^2^ = 0.767), the group × time interaction was not significant (*p* = 0.141). This indicates that the improvement in FVC was largely due to time-dependent adaptations (Table 7 and Figure 5).

In contrast, the increase in FEV_1_ values in the HG (15.89%) was higher than in the CG (10%), and both the time effect (F = 98.288, *p* < 0.001, η*p*^2^ = 0.845) and the group × time interaction (F = 6.620, *p* = 0.019). This result supports that the improvement in airway function is intervention-specific (Table 7 and Figure 6).

Significant improvements in favour of the HG were observed in peak expiratory flow (PEF) and maximal voluntary ventilation (MVV) parameters. The time effect was quite strong for both parameters (PEF: η*p*^2^ = 0.865; MVV: η*p*^2^ = 0.791), and the group × time interactions were significant (PEF: *p* = 0.002; MVV: *p* < 0.001). These findings indicate that the intervention significantly improved ventilatory capacity and expiratory flow rates (Table 7, Figure 7 and Figure 8).

A 24% increase in performance was observed in the HG in the Yo-Yo Intermittent Recovery Test Level 1 (Yo-Yo IRT1), while this increase was limited to 15.53% in the CG. The time effect was significant (F = 83.483, *p* < 0.001, η*p*^2^ = 0.763) and the group × time interaction was also statistically significant (F = 5.190, *p* = 0.031), indicating that the intervention improved intermittent high-intensity exercise performance (Table 7 and Figure 9).

## 4. Discussion

The primary aim of this research was to investigate the impact of post-training halotherapy, conducted in an artificial salt cave, on the performance parameters of female football players competing in the first division. The main findings of this study indicate that post-training halotherapy in elite female football players resulted in positive adaptations with significant and large effect sizes on respiratory muscle strength, pulmonary function, and aerobic performance. Notably, the marked increases in maximal inspiratory and expiratory pressure (MIP, MEP), VO_2_max, ventilatory capacity (MVV), and Yo-Yo IRT1 performance suggest that halotherapy may play a complementary role in the cardiorespiratory system in addition to traditional training stimuli. Considering that the literature has primarily focused on the respiratory function-improving effects of halotherapy in clinical and pediatric populations, these findings are unique and important in that they reveal its effects on performance-related respiratory parameters in elite team sport athletes. The results obtained suggest that halotherapy can be considered not only as a therapeutic method that supports respiratory health but also as a potential ergogenic aid that can contribute to performance optimization in sports requiring high aerobic and intermittent performance.

By shedding light on the potential benefits of halotherapy for aerobic performance in female football players, this study adds valuable insights to the field of sports science. It offers implications for optimizing training strategies to enhance athletic performance. The present study found that the halotherapy group showed improvements in FEV1, VO_2_max (mL/kg/min), and Yo-Yo IRT1 (m) compared with the control group. This indicates that the positive effects of halotherapy were statistically significant and manifested as differences between the two groups. The findings suggest that the halotherapy intervention improved performance parameters in female football players, particularly respiratory and aerobic capacities. However, further research is needed to elucidate the mechanisms underlying halotherapy and to optimize its use as a complementary method to enhance sports performance [20].

Consequently, utilizing halotherapy as an innovative recovery and conditioning modality could potentially optimize Yo-Yo IRT1 performance, thereby sustaining the physiological demands of elite football players during match conditions. Corroborating these theoretical frameworks, the present study demonstrates that the halotherapy intervention yielded statistically significant improvements in FEV1, VO_2_max, and Yo-Yo IRT1 (m) performance compared to the control group [36,42,43,44,45]. These findings provide empirical evidence that halotherapy significantly enhances the respiratory efficiency and aerobic capacity of elite female football players, thereby offering novel insights into the field of sports science. Consequently, integrating controlled microclimate exposures into periodized training regimens may serve as an effective strategy to optimize athletic performance and accelerate inter-effort recovery. Nevertheless, further mechanistic investigations are warranted to fully elucidate the underlying physiological pathways of dry saline aerosols and to establish standardized protocols for their application as a complementary ergogenic aid in high-performance sports.

The Yo-Yo IRT1 running performance observed in this study for female football players (ranging from 1307 to 1736 m) was similar to that reported in other studies of elite female football players [46,47]. This suggests that the participants in the current study performed at a level comparable to that of elite players in terms of aerobic capacity and endurance during high-intensity running. The study by Antonovici et al. [47] highlights the potential benefits of aerosolized air as a therapeutic intervention for improving various aspects of physical health. By optimizing cardiovascular efficiency, enhancing body resistance, and promoting muscle development, aerosolized air inhalation therapy may offer a promising approach to supporting individuals’ overall well-being and physical performance. Upon examining the study results, it is evident that the Yo-Yo IRT1 and VO_2_max levels of the halotherapy group improved more than those of the control group. This difference in improvement is attributed to increases in lung function and respiratory parameters, such as FEV, MVV, PEF, MIP, and MEP. The FEV, MVV, PEF, MIP, and MEP are key indicators of lung function and respiratory health. Improvements in these parameters suggest enhanced lung capacity and respiratory efficiency. In previous studies, Halotherapy has been observed to improve body composition, well-being, respiratory parameters, and lung capacity [48,49,50]. By enhancing respiratory efficiency, halotherapy may have contributed to the greater improvements in Yo-Yo IRT1 performance and VO_2_max levels in the halotherapy group, as observed in this study. Improvements in Yo-Yo IRT1 performance are particularly meaningful because this test has been shown to correlate with the amount of high-intensity running performed during competitive football matches. Consequently, the observed increase in Yo-Yo IRT1 distance suggests that halotherapy may contribute to improved match-related endurance and repeated high-intensity exercise capacity.

An interesting finding of the present study was that significant Group × Time interactions were observed for FEV_1_, PEF, MVV, MIP, and MEP, whereas no additional effect was detected for FVC. This observation is physiologically plausible because FVC primarily reflects static lung volume, which is largely determined by anatomical characteristics and is less responsive to short-term interventions in healthy, well-trained athletes. In contrast, FEV_1_, PEF, MVV, MIP, and MEP are functional indices of respiratory muscle performance, ventilatory capacity, and expiratory airflow and are therefore more sensitive to improvements in respiratory efficiency. Accordingly, the absence of a significant additional effect on FVC does not contradict the observed improvements in the other respiratory parameters but rather suggests that halotherapy may predominantly influence functional respiratory performance rather than static lung volume.

The study by Chervinskaya [51] demonstrated that halotherapy yielded positive effects on primary clinical outcomes, particularly in respiratory function, including cough and sputum characteristics. The positive impact of halotherapy was attributed to its effects on local defense mechanisms, resulting in increased resistance of the mucous membranes and enhanced secretion of secretory IgA (SIgA). Additionally, halotherapy has been shown to reduce colonization by pathogenic microorganisms, thereby improving respiratory health. In addition to its benefits for local defense mechanisms, halotherapy may influence the respiratory system, potentially improving respiratory rate during exercise. When individuals exercise, their respiratory muscles increase oxygen uptake. By enhancing respiratory function through halotherapy, breathing frequency can decrease during exercise, thereby allowing more efficient oxygen uptake by the blood. This improvement in lung capacity achieved through halotherapy is believed to contribute to better Yo-Yo IRT1 performance. The findings suggest that halotherapy can positively affect both the respiratory system’s functional characteristics and its immune defense mechanisms, thereby enhancing aerobic capacity and endurance. By improving lung function and reducing respiratory rate, halotherapy may confer a competitive advantage on athletes during high-intensity activities, such as those encountered in football matches.

The physiological mechanism underlying the effect of Halotherapy on aerobic performance and respiratory parameters can be explained as follows: the physical properties of haloaerosol promote the formation of respirable particles, enabling it to reach the deepest regions of the bronchial tree. This haloaerosol has a high surface energy, leading to a negative charge on the particles. This characteristic induces a stimulation effect (mucokinetic effect) on the cilia/spurs in the respiratory epithelium. Additionally, when inhaled, the dry salt particles in the haloaerosol condense within the mucus present in the bronchial tree, resulting in a mucolytic effect. This process facilitates mucus dilution and acts as an absorbent, effectively removing foreign substances from the respiratory system, much like a sponge. Consequently, this promotes mucus expulsion through coughing, facilitated by an increase in expiratory flow rate. The mucokinetic effect and the aforementioned mechanisms lead to several positive outcomes. Firstly, they contribute to the improvement of inflammatory processes in the bronchi and enhance mucosal immunity. Secondly, they help reduce bronchial obstruction and prevent bronchial remodeling. Furthermore, the use of halotherapy increases respiratory capacity and improves lung ventilation. These combined effects also translate into improvements in various performance indicators. The study by Horlenko et al. [52] elucidates the beneficial effects of halotherapy on the respiratory system. By understanding how haloaerosol affects the respiratory epithelium and facilitates the removal of mucus, foreign substances, and inflammatory factors, researchers and practitioners can better appreciate the potential applications of halotherapy for respiratory conditions and athletic performance. These findings suggest that halotherapy may enhance respiratory health, with possible implications for athletes’ aerobic capacity and overall performance. Regarding another mechanism, it has been suggested that salty air may increase VO_2_max (maximum oxygen consumption, a measure of aerobic capacity). The researchers further proposed that this increase in VO_2_max is related to both systemic oxygen extraction and the content of the salty air. They also indicated that delivering oxygen to the active muscles of endurance-trained athletes via salty air could increase VO_2_max, which may be associated with the skeletal muscle’s internal mitochondrial respiratory capacity [52]. The observed improvements in VO_2_max and respiratory function may be consistent with enhanced respiratory efficiency. Beyond statistical significance, the observed improvements appear to be practically relevant for elite female football players. To our knowledge, universally accepted minimal clinically important difference (MCID) values have not yet been established for these outcomes in elite female football players. Therefore, the practical relevance of our findings was interpreted using effect sizes, percentage changes, and their known relationships with football-specific performance reported in previous literature. In football, even modest enhancements in aerobic capacity and respiratory function may improve the ability to perform repeated high-intensity efforts, accelerate recovery between intense actions, and maintain technical performance during the later stages of a match. To our knowledge, universally accepted minimal clinically important differences have not yet been established for these physiological outcomes in elite female football players. Therefore, the physiological adaptations observed in the present study may have meaningful implications for competitive performance, particularly during the preseason preparation period. Nevertheless, the present study did not evaluate oxygen extraction, mitochondrial function, inflammatory biomarkers, or respiratory muscle activation. Therefore, these mechanisms remain speculative and require confirmation in future mechanistic studies.

**Limitations**

When interpreting the findings of this study, certain methodological limitations must be taken into account. First, participants were recruited from a single professional women’s football club, which may limit the external validity of the results due to club-specific training practices and environmental factors. Although power analysis supports the statistical adequacy of the study, the sample consisting of a single team and athletes with a homogeneous performance level limits the generalizability of the results to different league levels, age groups, and male athletes. Second, the study was conducted only during the preseason period. The sustainability of halotherapy’s effects during the season or in long-term applications, or its impact on performance fluctuations, was not evaluated within the scope of this study. This limits the ability to draw conclusions about the chronic effects of halotherapy and its applicability throughout the season. Third, the athletes’ dietary habits, daily energy intake, and hydration status were not systematically monitored during the study peri-od. As these factors can affect aerobic performance and respiratory function, they may constitute a limiting factor in attributing the observed adaptations solely to halotherapy. Fourth, the sample consisted exclusively of elite Turkish female football players; therefore, caution is warranted when generalizing these findings to male players, youth athletes, recreational populations, or athletes from different competitive levels or cultural backgrounds. Fifth, no long-term follow-up assessments were performed after completion of the intervention. Consequently, the persistence of the observed improvements in respiratory function and aerobic performance remains unknown. Future multicentre randomized controlled trials including athletes from different clubs, competitive levels, and countries, together with long-term follow-up assessments, are needed to confirm the generalizability and durability of these findings. Additionally, menstrual cycle phases, which could affect performance and respiratory physiology in female athletes, were not controlled. Considering the potential effects of hormonal fluctuations on respiratory parameters and aerobic capacity, this situation can be considered a potential limitation in interpreting the results. The study did not evaluate perceptual and psychological variables related to the effects of halo-therapy (subjective measures such as perceived recovery, respiratory comfort, or exercise comfort). Therefore, the extent to which physiological improvements corresponded with the athletes’ subjective experiences could not be determined. An important methodological limitation of the present study is the absence of a sham-halotherapy control condition. Because participants in the halotherapy group were aware that they were receiving an additional intervention, expectancy and placebo effects cannot be completely excluded. Although both groups followed identical preseason training programs and objective physiological outcomes were assessed, future randomized controlled trials should incorporate sham-halotherapy conditions that replicate the environmental characteristics of the halochamber without delivering therapeutic salt aerosol. Such designs would allow clearer separation of the physiological effects of halo-therapy from potential placebo responses. Although internal training intensity was monitored using session-RPE and maintained within the target range of 5–7 AU, cumulative weekly training load (e.g., session duration × sRPE), heart-rate responses, and GPS-derived external load variables were not systematically quantified. Therefore, subtle between-group differences in total physiological training load cannot be completely excluded. Finally, biochemical or cellular markers that could directly explain the physiological mechanisms underlying respiratory adaptations (e.g., inflammatory markers, oxidative stress parameters, or electromyographic measurements of respiratory muscle activation) were not examined in the study. This limits the mechanistic interpretation of halotherapy’s. In addition, body composition variables, such as body fat percentage, were not assessed. Therefore, the potential influence of halotherapy on body composition could not be evaluated. Future randomized controlled trials should incorporate validated body composition assessments to provide a more comprehensive evaluation of the intervention.

**Practical Implications**

Based on the findings of the present study, halotherapy may be considered as an adjunctive recovery and respiratory support strategy for elite female football players during the preseason period. The intervention was administered three times per week for 12 weeks before scheduled football training sessions and was associated with improvements in respiratory muscle strength, pulmonary function, aerobic capacity, and intermittent running performance. Given the time constraints faced by professional teams, halotherapy provides a non-fatiguing intervention that can be used during recovery sessions. Based on the protocol in this study, a minimum weekly commitment of about 30 min after training, 2–3 times per week, might be enough to see measurable improvements. These findings suggest that integrating halo-therapy into preseason conditioning programs may provide a practical adjunct to conventional football training by improving respiratory muscle strength, pulmonary function, and aerobic fitness. Although universally accepted MCID values are currently unavailable for these outcomes in elite female football players, the observed improvements, together with their moderate-to-large effect sizes, indicate that the intervention may have meaningful practical value in supporting match-related endurance and repeated high-intensity performance. Therefore, coaches and sports medicine practitioners may consider incorporating halotherapy into preseason conditioning programs when the primary goal is to optimize respiratory function and aerobic fitness. However, because the present findings were obtained exclusively in elite Turkish female football players during the preseason, further research is required before similar recommendations can be extended to other athletic populations, competitive levels, or phases of the season.

**Future research**

Future research should examine optimal dosing, timing related to training and competition phases, and whether combining halotherapy with other recovery methods results in synergistic benefits. It should also include male athletes and different age groups to evaluate sex- and age-specific responses, monitor dietary intake, hydration, and menstrual cycle phases to control confounding variables, and investigate psychological and subjective effects of halotherapy. Exploring underlying mechanisms through physiological and biochemical markers and conducting long-term studies throughout entire competitive seasons are also recommended.

## 5. Conclusions

This study demonstrated that a 12-week halotherapy intervention administered during the preseason period produced positive adaptations with significant and large effect sizes on respiratory muscle strength, pulmonary function, aerobic capacity, and Yo-Yo IRT1 running performance in elite female football players. The improvements observed in the halotherapy group in maximal inspiratory and expiratory pressure (MIP and MEP), FEV_1_, PEF, MVV, VO_2_max, and Yo-Yo IRT1 performance were determined to be beyond the gains associated with regular football training alone. Although an increase in forced vital capacity (FVC) was observed over time in both groups, the lack of a significant group × time interaction suggests that this change is largely due to general training adaptations. In contrast, the marked increases in respiratory muscle strength, expiratory flow rates, and maximal ventilation capacity, in particular, suggest that halotherapy may play a complementary role in ventilatory efficiency and respiratory mechanics. The findings of the present study suggest that halotherapy may represent a promising complementary strategy for enhancing respiratory function and aerobic performance in elite female football players during the preseason. However, given the single-club design, the exclusive inclusion of elite Turkish female players, and the absence of long-term follow-up, these findings should be interpreted with caution. Confirmation through larger multicentre randomized controlled trials involving diverse athletic populations is warranted before broad recommendations can be made.

## Figures and Tables

**Table 1:** Descriptive data for the control and halotherapy group.

Variables	Control (n:14)	Halotherapy Group (n:14)	*p*
Age (year)	21.93 ± 2.13	23.14 ± 2.63	0.125
Height (cm)	169.86 ± 5.30	165.96 ± 5.09	0.059
Weight (kg)	59.96 ± 3.34	62.63 ± 3.02	0.441
VO_2_max	47.39 ± 1.69	47.45 ± 0.96	0.906

**Table 2:** Study design.

	Week 1	Weeks 2–13	Week 14
**Activity**	Pre-intervention testing	Intervention period (halotherapy for HG + training)	Post-intervention testing

**Table 3:** Weekly Training Structure.

	Sessions per Week	Sessions per Week
Endurance & aerobic drills	3	Interval running, continuous running
Technical & tactical training	4	Ball control, passing, and team strategies
Strength training (gym)	2	Resistance exercises focused on the lower and upper body

**Table 4:** Progression of the preseason training programme across the 12-week intervention period.

Weeks	Primary Training Focus	Target Intensity (sRPE)
1–2	General adaptation and aerobic conditioning	5–6 AU
3–5	Aerobic development and technical/tactical training	5–6 AU
6–8	Strength development and repeated-sprint training	6–7 AU
9–10	High-intensity match-specific conditioning	6–7 AU
11–12	Performance optimization and preseason preparation	5–6 AU

**Table 5:** Timeline of the study.

Days	1st Week	From 2nd to 13th Week	14th Week
Sunday	Adaptation training	Resting day	Resting day
Monday	Adaptation training	HS + football training	football training
Tuesday	Adaptation training	football training	Resting day
Wednesday	Pre-test data collection	HS + football training	Post-test data collection
Thursday	Resting day	football training	football training
Friday	Resting day	HS + football training	football training
Saturday	Resting day	Resting day	Resting day

**Table 6:** Concentration of dry sodium chloride aerosol and duration of halotherapy.

	Parameter	Value/Description
Session frequency	3 sessions/week	Before training sessions
Session duration	45 min	Individual treatment in reclining chairs
Aerosol concentration	20 mg/m^3^	Constantly monitored by a halogen generator
Particle size	<5 microns	>80% respirable fraction
Environmental controls	Temperature: 20–24 °C; Humidity: 40–60%	Automated lighting and ventilation adjustments
Additional elements	Relaxing music and visuals	To promote athlete comfort and relaxation

**Table 7:** Changes in respiratory muscle strength, pulmonary function, and aerobic performance before and after the intervention in the Halotherapy and control groups.

Parameters	Group	Pre-Test Mean ± SD	95% CI		Post-Test Mean ± SD	95% CI		Δ%	Time Effect		Group × Time Interaction	
			LB	UB		LB	UB		F	*p* (η*p*^2^)	F	*p* (η*p*^2^)
MIP (cmH_2_O)	HG	76.46 ± 2.22	74.24	78.68	101.78 ± 5.29	96.49	107.07	33.11	217.522	<0.001 (0.924)	61.790	0.014 (0.774)
	CG	78.33 ± 0.94	77.39	79.27	86.05 ± 2.50	83.55	88.55	9.85				
MEP (cmH_2_O)	HG	89.23 ± 1.44	87.79	90.67	108.21 ± 4.41	103.8	112.62	21.27	23.544	<0.001 (0.523)	6.857	<0.001 (0.341)
	CG	88.83 ± 1.29	87.54	90.12	96.67 ± 3.51	93.16	100.18	8.82				
VO_2_max (mL/kg/min)	HG	47.45 ± 0.96	46.69	48.21	51.74 ± 1.06	50.85	52.62	9.04	136.569	<0.001 (0.840)	25.535	<0.001 (0.495)
	CG	47.39 ± 1.69	46.63	48.14	49.08 ± 2.02	48.20	49.97	3.57				
FVC (L)	HG	4.61 ± 0.15	4.46	4.76	5.09 ± 0.14	4.95	5.23	10.41	59.259	<0.001 (0.767)	2.370	0.141 (0.116)
	CG	4.35 ± 0.20	4.15	4.55	4.67 ± 0.18	4.49	4.85	7.35				
FEV_1_ (L)	HG	4.34 ± 0.13	4.21	4.47	5.03 ± 0.09	4.94	5.12	15.89	98.288	<0.001 (0.845)	6.620	0.019 (0.269)
	CG	4.10 ± 0.17	3.93	4.27	4.51 ± 0.28	4.23	4.79	10				
PEF (L/min)	HG	7.68 ± 0.20	7.48	7.88	8.81 ± 0.36	8.45	9.17	14.71	115.731	<0.001 (0.865)	12.647	0.002 (0.413)
	CG	7.85 ± 0.17	7.68	8.02	8.41 ± 0.24	8.17	8.65	7.13				
MVV (L/min)	HG	110.38 ± 1.15	109.23	111.53	114.59 ± 1.01	113.58	115.6	3.81	68.026	<0.001 (0.791)	26.915	<0.001 (0.599)
	CG	110.02 ± 1.59	108.43	111.61	110.98 ± 1.47	109.51	112.45	0.87				
Yo-Yo IRT1(m)	HG	1400 ± 157.68	1242.32	1557.68	1736.43 ± 162.32	1574.11	1898.75	24	83.483	<0.001 (0.763)	5.190	0.031 (0.166)
	CG	1307.86 ± 201.54	1106.32	1509.4	1510 ± 240.22	1269.78	1750.22	15.53				

## Data Availability

The data used to support the findings of this study are available from the corresponding author upon request.

## Supplementary Materials

The following supporting information can be downloaded at https://www.mdpi.com/article/10.3390/life16081233/s1. Reference [53] is cited in the supplementary materials.
